# Assessment of the reproducibility and precision of milling and 3D printing surgical guides

**DOI:** 10.1186/s12903-020-01362-6

**Published:** 2021-01-02

**Authors:** Sueli Mukai, Eduardo Mukai, José Arnaldo Santos-Junior, Jamil Awad Shibli, Marcelo Faveri, Gabriela Giro

**Affiliations:** 1 Dental Research Division, Department of Periodontology and Oral Implantology, Guarulhos University, Praça Tereza Cristina 289, Guarulhos, SP 07030-070 Brazil; 2 Private Practice, Av. Conselheiro Carrão, 1530, São Paulo, SP 03402-001 Brazil

**Keywords:** Surgical guide, Milling surgical guide, 3D printing surgical guide, Virtual guided surgery, CAD/CAM

## Abstract

**Background:**

Technology advancement has rising in the past decade and brought several innovations and improvements. In dentistry, this advances provided more comfortable and quick procedures to both the patient and the dental surgeon, generating less predictability in the final result. Several techniques has been developed for the preparation of surgical guides aiming at the optimization of surgical procedures. The present study aimed to evaluate the reproducibility and precision of two types of surgical guides obtained using 3D printing and milling methods.

**Methods:**

A virtual model was developed that allowed the virtual design of milled (n = 10) or 3D printed (n = 10) surgical guides. The surgical guides were digitally oriented and overlapped on the virtual model. For the milling guides, the Sirona Dentsply system was used, while the 3D printing guides were produced using EnvisionTEC’s Perfactory P4K Life Series 3D printer and E-Guide Tint, a biocompatible Class I certified material. The precision and trueness of each group during overlap were assessed. The data were analyzed with GraphPad software using the Kolmogorov–Smirnov test for normality and Student’s *t* test for the variables.

**Results:**

The Kolmogorov–Smirnov test showed a normal distribution of the data. Comparisons between groups showed no statistically significant differences for trueness (*p* = 0.529) or precision (*p* = 0.3021). However, a significant difference was observed in the standard deviation of mismatches regarding accuracy from the master model (*p* < 0.0001).

**Conclusions:**

Within the limits of this study, surgical guides fabricated by milling or prototyped processes achieved similar results.

## Background

Rehabilitating a patient with an implant requires precise planning and special care during surgery. Placing a poorly planned implant can cause real problems, such as the perforation of critical anatomical structures, increased surgical duration, patient anxiety, pain, and stress. Therefore, presurgical planning using instruments such as tomography and surgical guides is essential [1–3].

The use of surgical guides in dentistry has provided patients and dental surgeons with greater flexibility, accuracy, and control of the procedure being executed [4, 5], resulting in a more comfortable postoperative experience for the patient and, by reverse planning, delivery of the immediate prosthesis or optimization of the final prosthetic result [6–8]. Different types of guides may be used during surgery. Conventional surgical guides are made of acrylic resin that, unfortunately, does not provide the crucial anatomical information needed for the surgical procedure. To carefully position the implants, avoid bone augmentation procedures, and optimize the surgical procedure, cone-beam computed tomography (CBCT)-guided implant surgery has been the best option because implants inserted using virtually guided procedures are more precise than those involving conventional procedures [9–11]. The surgical guide for CBCT-guided surgery is made using a combination of software, which, together with CBCT, transfers anatomical data for the presurgical planning of the implant [2, 9, 10]. These guides create a combination of systems that integrate tomography and chairside CAD/CAM to optimize and simplify planning from the first consultation through implant installation [13]. This technology has revolutionized dentistry by allowing the dental surgeon to generate the surgical guide using a completely virtual approach and plan the surgery so that implants are inserted based on available bone, thereby reducing the surgery’s duration and possibility of complications [14, 15]. Several authors have investigated different materials to evaluate the implant positioning and the deviations of the implant along its body using various techniques [16]. Other factors could influence implant positioning, such as practitioner experience, surgical approach, and tissue support [17–19]. The literature shows that using materials that allow higher flexure or deformations may increase implant positioning deviations [16], particularly in the edentulous space with multiple missing teeth.

The technology developed to produce these surgical guides is an innovative system. However, its use remains restricted because few studies are available on the production and use of this type of procedure, making new studies a priority. Therefore, this study aimed to evaluate and compare the reproducibility and precision of milled and three-dimensional (3D) printing surgical guides compared with the initial virtual project. The null hypothesis is that both milling and 3D printing make surgical guides plausible for use in guided surgery.

## Methods

This study used a partially edentulous area Kennedy class IV model to compare both groups. The sample size was calculated using the Sample Calculator (https://www.calculator.net/sample-size-calculator.html), with the lower discrepancy value between groups, and the number of samples required for the test was 10. Therefore, the surgical guide was reproduced ten times for the evaluations.

The same model selected for the study was used to create all surgical guides was divided into two distinct groups: the MILLED GROUP, comprising 10 milled surgical guides and 3D PRINTING GROUP, comprising 10 3D printing surgical guides.

### Production of surgical guides

The model used in this study was digitized using an intraoral scanning system (Cerec AC^®^; Sirona Dentsply, Bensheim, Germany). The scanning process generated a projection in SSI language that enabled virtual planning of the ideal position for inserting the implant. After that, an image was developed in DXD language, allowing file import to inLab 15 software (Sirona Dentsply, Germany), making it possible to create the appropriate design of the surgical guide.

**Fig. 1:**
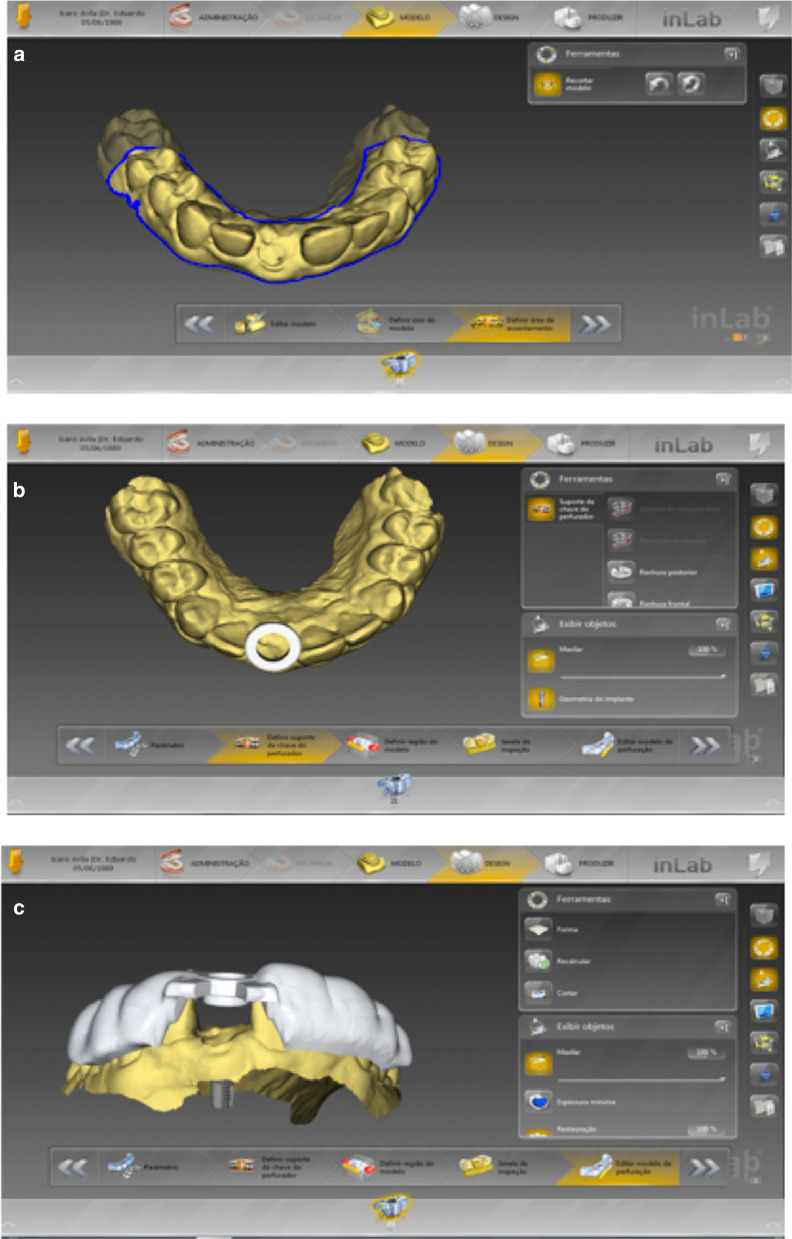
**a** Definition of the limits of the surgical guide. **b** Ring position. **c** Projection of the final surgical guide

The surgical guide design started by defining the ridge boundaries and length of the surgical guide (Fig. 1a) and determining the ring's position and size responsible for guiding implant insertion (Fig. 1b). After this step, a preview of the surgical guide design was generated (Fig. 1c). After verification and approval of the planned guide, the project was ready for manufacture.

For the milling guides, the file was sent to the MCXL milling machine (Sirona Dentsply, Germany) for production using polymethyl methacrylate (PMMA) according to the manufacturer’s standardized parameters.

3D printing guides were produced after converting the DXD archive into STL extension, which was then sent to a 3D printer (Perfactory P4K Life Series, EnvisionTEC, Germany). This printer uses DLP technology with a 4-M pixel projector and a UV wavelength of 385 nm. The printer was calibrated following the manufacturer’s instructions before the beginning of the printing process (detailed instructions can be found on page 31 of the printer’s technical guide). The resin used was EnvisionTEC's E-Guide Tint (Dearborn, USA), which is a biocompatible Class I certified material. The guides were positioned at a 45° angle. After printing, the guides were immersed in isopropyl alcohol to perform surface cleaning, and then light curing was performed using the manufacturer’s parameters.

**Fig. 2:**
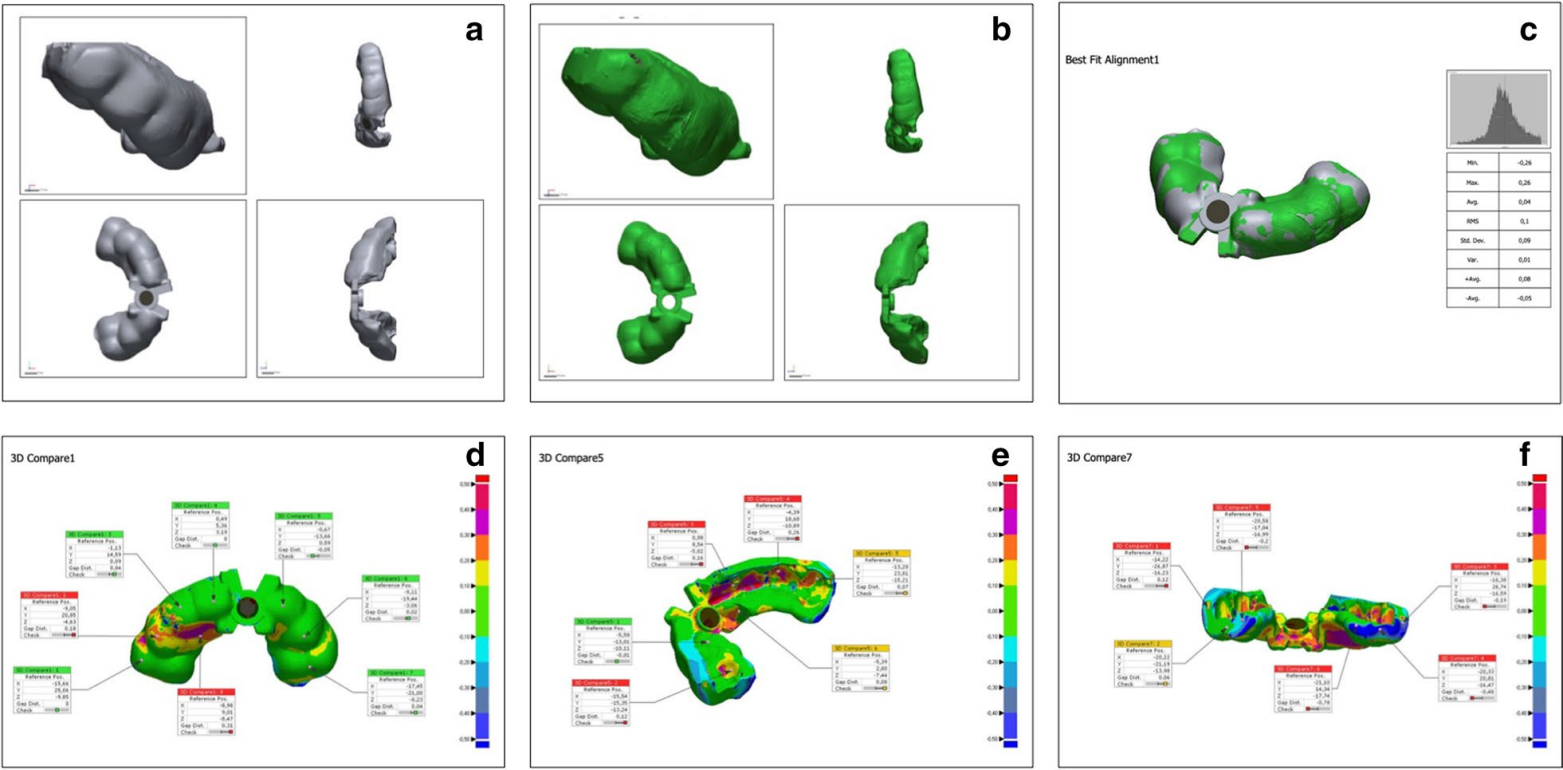
Representative sample of the comparison between the virtual guide and test guide after the best superimposition on the master model was achieved. **a** Virtual surgical guide generated in the master model. **b** 3D printing surgical guide. **c** Best fit alignment of the superimposition of both guides. **d** Front view of the comparisons between guides. **e** Inside view of the best fit alignment comparison between guides. **f** Back view of the surgical guide comparison

Once the surgical guides were completed, individual digitization of each surgical guide was performed using a Data Sheet camera (stereoSCAN^3D^ R8; 8.0 megapixel, Germany), thereby creating a mathematical model (STL) so that the guides could be superimposed overlapping the virtual master model using the software Optocat (Breuckmann, Heiligenhaus, Germany). The sequence is illustrated in Fig. 2.

Once the best fit alignment between images was obtained, the superimposed models were evaluated and the areas of misalignment were identified. The data obtained with the superimposed files were evaluated between the groups for the precision in obtaining the guides from the master model (intergroup evaluation). Additionally, the guides of the same group were compared, obtaining the trueness to verify the reproducibility of the guides using both fabrication processes (intragroup evaluation).

The minimal and maximum values of misalignment for each group, the average of a mismatch for each sample, and the standard deviation between these misalignments in each model were recorded.

### Data analysis

The data were evaluated using GraphPad software. The Kolmogorov–Smirnov test was performed to test the normality of the data for precision (> 0.1000 and 0.0637 for the milled and 3D printing groups, respectively) and trueness (0.571 and > 0.1000 for the milled and 3D printing groups, respectively). After passing the normality test, the data were submitted to the parametric evaluation of Student’s *t*-test. The alpha level for significance was set at 5%.

## Results

**Fig. 3:**
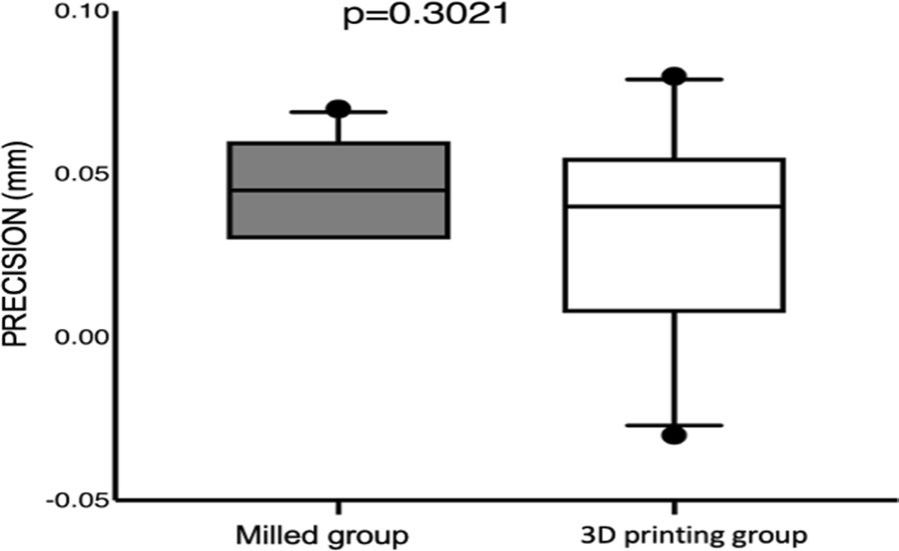
Graphical representation for the average of mismatches for both groups regarding the best fit alignment on the master model (precision evaluation)

**Fig. 4:**
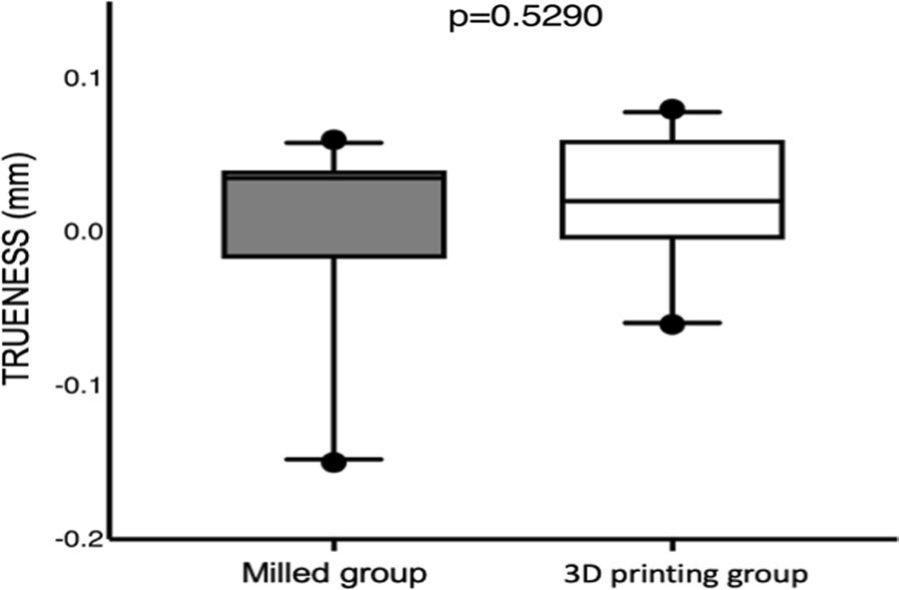
Graphical representation for the average of mismatches for both groups regarding the reproducibility of the guides (trueness evaluation)

The comparisons between groups for precision and trueness are presented in Tables 1 and 2, respectively. No statistically significant differences between the groups were observed for the average of a mismatch for both precision (*p* = 0.302) and trueness (*p* = 0.529), showing that superimposing the master model or evaluating the reproductivity of the guides were similar for the milling and 3D printing processes. However, the variation was greater in the 3D printing process for precision evaluation because the standard deviation of the misalignments presented higher scores than those in the milling group (*p* < 0.0001). The data distribution could be better observed in Figs. 3 and 4 for precision and trueness, respectively, because the boxplot graphs represent the median, 25% and 75% quartiles and maximum and minimum values for the average mismatch of each group.

**Table 1:** Data for PRECISION evaluation between milling and 3D printing groups. It can be observed the minimum and maximum values of mismatch found in each group, and the average mismatch and the average for each group of the standard deviation found in each sample

PRECISION	Minimun value	Maximum value	Average mismatch (mm)	SD
Milling guide	− 0.18	0.2	0.0484	0.04
3D printing guides	− 0.48	0.48	0.034	0.112
			0.3021	< 0.0001

**Table 2:** Data for TRUENESS evaluation between milling and 3D printing groups. It can be observed the minimum and maximum values of mismatch found in each group, and the average mismatch and the average for each group of the standard deviation found in each sample

TRUENESS	Minimun value	Maximum value	Average mismatch (mm)	SD
Milling guide	− 5	5	0.002	0.467
3D printing guides	− 5	4.8	0.02	0.37
*p* value			0.529	0.2912

## Discussion

This study's objective was to compare two different surgical guides in terms of their reproducibility and precision relative to the initial virtual projection. Despite being an in vitro study, this study was the first to compare the accuracy of the reproductivity of the main methods used to fabricate surgical guides in the era of digital dentistry. This evaluation was performed by superimposing images, a procedure that allows the point-by-point evaluation of any discrepancy in the guide characteristics. No difference was found regarding the best fit alignment between the groups, suggesting that both the milling and printing fabrication methods are suitable for use with good reproductivity in guided surgeries. Park et al. [2] observed that milled surgical guides had less deviation than 3D printing guides (*p* < 0.05). Moreover, other authors have reported greater precision for milled guides regarding the final implant position [10, 12, 15, 20]. However, clinically, this error does not seem to influence the final result of the rehabilitation. Bell et al. [16] evaluated two different surgical guide materials concerning the implant’s angular deviation inserted using thermoplastic and 3D-printed surgical guides. The authors demonstrated no clinical difference between the groups, although the implants placed using the thermoplastic surgical guide were less accurate on apex positioning.

The results of the other studies comparing the final implant position obtained using both guides are questionable because various factors can influence the precision of guided surgery, such as scanning errors, errors in producing the guides, mechanical errors, data transmission errors, and human error [8, 10, 12, 17, 21]. These factors are cumulative and interactive and can occur at any time during the process. In this study, we showed that the reproduction of guides based on the same scan of the same model, using CAD/CAM-assisted surgical guides, is a technique as precise as that using 3D printing guides. This topic remains controversial in the literature because some authors have shown the advantages of using 3D printing over conventional surgical guides produced on top of models and over implants that are positioned freehand [9, 10, 19, 22] while other studies have shown no significant difference in the implant survival rate and effectiveness using conventional or digital implant placement procedures [11].

The literature also shows that 3D printing surgical guides may be associated with surgical complications caused by problems during their production. These problems include a lack of calibration of the printing equipment, changes to the physical properties of the resin, difficulty in positioning or fixing the guide in the oral cavity, or limitations in mouth opening [7, 9, 23]. It is essential to know the prototyping guide technique's limits to minimize complications during the surgical procedure. Van Assche et al. [1] observed that to avoid deforming 3D printing guides, it is essential that the guide has a total thickness of 2.5 to 3.0 mm. This deformity is not observed in milled guides because the resin blocks are ready to be machined without changing their structure [12].

Despite the precision found in this study’s results, the literature suggests that errors may occur during the manufacture of either type of surgical guide. Thus, it is recommended that a 2-mm safety margin be maintained around important and vital structures [2, 21] and that cone-beam computed tomography images be used to achieve a correct evaluation of the essential anatomical structures [9, 20, 24, 25].

Clinically, the goal of precise surgical guides is to avoid damaging the noble structures and offer an ideal treatment plan that meets the patient’s aesthetic and functional objectives [3, 15], with a shorter duration of surgery and fewer complications during surgery. Although the results of this study showed no differences in reproducibility and precision for the different methods of generating surgical guides, future studies are needed to gauge the implications that such differences may have on surgical positioning. It is also necessary to evaluate the cost/benefit ratio of both types of guides for the patient and dental surgeon.

## Conclusion

According to the results obtained in this study, it is suggested that no difference is observed in the degree of mismatch during overlapping when comparing 3D printing surgical guides and milled guides for precision and trueness evaluations.

## Data Availability

The datasets analyzed during the current study are available from the corresponding author.

## Abbreviations

CBCT: Cone-beam computed tomography
3D printing: Three-dimensional printing
CAD/CAM: Computer-aided design and computer-aided manufacturing
DXD and STL language: Mathematical model computer software
